# Patient Experiences of Rehabilitation and the Potential for an mHealth System with Biofeedback After Breast Cancer Surgery: Qualitative Study

**DOI:** 10.2196/19721

**Published:** 2020-07-29

**Authors:** Louise Brennan, Threase Kessie, Brian Caulfield

**Affiliations:** 1 Physiotherapy Department Beacon Hospital Dublin Ireland; 2 Insight Centre for Data Analytics University College Dublin Dublin Ireland; 3 School of Public Health, Physiotherapy and Sports Science University College Dublin Dublin Ireland; 4 Maynooth University Innovation Lab Maynooth University Kildare Ireland

**Keywords:** breast cancer, physiotherapy, rehabilitation, mHealth, biofeedback, user-centred design, cancer

## Abstract

**Background:**

Physiotherapy-led home rehabilitation after breast cancer surgery can protect against the development of upper limb dysfunction and other disabling consequences of surgery. A variety of barriers can limit physical rehabilitation outcomes, and patients may benefit from more support during this time. Mobile health (mHealth) systems can assist patients during rehabilitation by providing exercise support, biofeedback, and information. Before designing mHealth systems for a specific population, developers must first engage with users to understand their experiences and needs.

**Objective:**

The aims of this study were to explore patients’ rehabilitation experiences and unmet needs during home rehabilitation after breast cancer surgery and to understand their experiences of mHealth technology and the requirements they desire from an mHealth system.

**Methods:**

This was the first stage of a user-centered design process for an mHealth system. We interviewed 10 breast cancer survivors under the two main topics of “Rehabilitation” and “Technology” and performed a thematic analysis on the interview data.

**Results:**

Discussions regarding rehabilitation focused on the acute and long-term consequences of surgery; unmet needs and lack of support; self-driven rehabilitation; and visions for high-quality rehabilitation. Regarding technology, participants reported a lack of mHealth options for this clinical context and using non-cancer–specific applications and wearables. Participants requested an mHealth tool from a reliable source that provides exercise support.

**Conclusions:**

There are unmet needs surrounding access to physiotherapy, information, and support during home rehabilitation after breast cancer surgery that could be addressed with an mHealth system. Breast cancer survivors are open to using an mHealth system and require that it comes from a reliable source and focuses on supporting exercise performance.

## Introduction

Breast cancer accounts for 1 in 4 cancer diagnoses in women in Europe [1]. A pattern of decreased mortality rates and increased incidence means that there is an ever-increasing number of women living with and beyond a breast cancer diagnosis [2]. The adverse side effects of breast cancer treatment can last for years after treatment finishes, limiting survivors’ quality of life and participation in activities of daily living [3,4]. Addressing the consequences of treatment in this growing population through rehabilitation is an essential component of the holistic management of this disease and an important goal for researchers and clinicians [5,6].

Upper limb dysfunction is a prevalent, persistent, and disabling consequence of breast cancer treatment, which is reported by up to 62% of women 6 years after treatment [3,7,8]. Symptoms such as shoulder girdle pain, weakness, reduced range of movement (ROM), and lymphedema are specifically associated with surgery for breast cancer [8-10]. Physiotherapy-led rehabilitation is recommended after breast cancer surgery to prevent development of upper limb dysfunction, to assist a return to full function, and to promote self-management and maintenance of healthy lifestyle behaviors [11,12]. A key component of physiotherapy rehabilitation is the home exercise program (HEP), which is usually prescribed to patients on the first day after surgery. Once discharged from hospital, the patient is expected to continue doing this rehabilitation exercise program daily at home without supervision from a physiotherapist, a process referred to as “home rehabilitation.” Performing exercise programs unsupervised at home can be challenging, and in the general population adherence rates to home exercise are as low as 65% [13]. In a breast cancer population there are additional barriers to rehabilitation to consider, such as the incapacitating side effects of cancer treatment, hospital admissions, and difficulty accessing physiotherapy in hospital or on discharge [14,15]. A large US-based survey by Reigle and Zhang [16] found that 61% of breast cancer survivors were instructed to perform upper body exercises after their surgery and only 28% received these instructions from a physiotherapist. Accessing a physiotherapist with experience in managing breast cancer–related upper limb impairments can be difficult, due to the low numbers of physiotherapists within this specialty [17]. Provision of a preoperative review and postoperative follow-up care for up to 1 year after surgery with long-term ongoing surveillance is recommended to optimize physical and functional well-being after breast cancer surgery [5,18,19]. Although access to physiotherapy services for breast cancer surgery varies between and within nations, it rarely meets these recommendations [20-22]. Considering the above barriers to rehabilitation, many women may benefit from more support after surgery to optimize their rehabilitation and limit the development of upper limb dysfunction.

The Global Observatory for electronic health (eHealth) defines mobile health (mHealth) as “medical and public health practice supported by mobile devices…and other wireless devices” [23]. MHealth apps can effectively support patients after surgery [24-27]. There is a promising role for mHealth apps and wearable sensors to enhance breast cancer rehabilitation by acting as motivational tools, to measure exercise interventions, and to support self-management [28-30]. Interventions that combine an app with a wearable external sensor can collect biomechanical or physiological data and feed this back to the user, allowing them to alter their exercise performance if needed; this process is called biofeedback [31,32]. Biofeedback during exercise is educational and motivational, can improve rehabilitation quality and safety, and can facilitate patient engagement [33-35]. Biofeedback mHealth systems can enable the collection of objective, longitudinal, and real-world data related to exercise performance in the home environment [30,36]. MHealth and biofeedback technologies have had an impact in several areas of breast cancer care, such as symptom management [37], screening [38], and general survivorship care [39]. However, these technologies are still in the very early stages of development and adoption in breast cancer rehabilitation. To date, innovations have mainly focused on physical activity interventions [40-42], lymphedema management [28], psychological well-being [43], and general survivorship care [29,44]. Very few interventions concentrate on physiotherapy rehabilitation exercises. There is a need to address this gap by developing an mHealth system that contains biofeedback features to support patients during physiotherapy rehabilitation in breast cancer.

To design an effective mHealth system, developers must first understand the experiences and unmet needs of the target population [45,46]. Unmet needs occur with a high incidence across the entire spectrum of breast cancer care [47,48]. Although some studies have investigated the unmet needs of patients within specific aspects of care, such as information needs [49,50] or psychosocial needs [51], to date, little is known about unmet needs during physiotherapy rehabilitation, despite its importance in reducing the adverse side effects of surgery. It is also essential in the early stages of digital technology development to understand the perspectives and experiences of the user in regard to the proposed technology [52,53]. Several studies have investigated breast cancer survivors’ attitudes to mHealth for general physical activity [54,55]; however, there is a need to understand their attitudes toward, experiences of, and requirements from mHealth technology for physiotherapy-led breast cancer home rehabilitation.

The aim of this study was therefore to explore the experiences and unmet needs of women during home rehabilitation following surgery for breast cancer and to gather survivors’ perspectives on and requirements from mHealth technology for postoperative breast cancer rehabilitation.

## Methods

### Study Design

A qualitative research study design using semistructured interviews with breast cancer survivors was undertaken to explore their experiences and needs regarding rehabilitation and technology. This methodology was chosen to allow a detailed description of experiences and opinions, which can produce rich, detailed, and complex data, even when collected from a small number of respondents [56,57]. The study was conducted and reported according to the Consolidated Criteria for Reporting Qualitative Research (COREQ) checklist for qualitative studies [58]. Ethics approval for this study was granted by University College Dublin Human Research Ethics Committee. All participants provided written informed consent to take part in this study.

### Participants and Recruitment

Participants for this study were women with a history of breast cancer living in the wider Dublin area, who were recruited from a sports club whose members are all survivors of breast cancer. This sample was chosen to ensure the data collected represented the general perspectives of breast cancer survivors and was not influenced by where they received cancer treatment. It was important to capture how consequences of surgery and postoperative rehabilitation may have impacted participants in the medium- and long-term [5]. Therefore, participants were individuals who had completed their treatment and could reflect on their experiences across and beyond the entire treatment pathway. Participants were required to be 18 years of age or older and to have had surgery for breast cancer within the last 5 years. This time limit served to ensure that current rehabilitation practices were represented, and to improve reliability of experience recollection. The lead researcher was introduced to the club members by a physiotherapist who is professionally associated with the club. Individuals interested in participating contacted the lead researcher, at which point they were screened for inclusion suitability. A sample size of 10 participants was chosen. This is in line with recommendations by Guest et al [59], who found that data saturation occurred within 12 interviews and meta-themes could be detected at 6 interviews. Other qualitative literature with breast cancer survivors used a similar number of participants [55,60,61].

### Data Collection

A literature review of unmet needs and technology in breast cancer rehabilitation, as discussed in the introduction to this study, informed the development of a flexible, semistructured interview guide, which is presented in Textbox 1. The interviews, which were audio-recorded, contained two main topics with two distinct aims: the first topic (questions 1.1-1.7) aimed to explore rehabilitation experiences and the second topic (questions 2.1-2.3) aimed to explore perspectives on, and opportunities for, mHealth during rehabilitation. The interview guide was developed in consultation with a postdoctoral researcher with expertise in qualitative data collection and analysis. Each participant had a one-on-one semistructured interview with the lead researcher (LB), who is a female physiotherapist and PhD researcher. Interviews lasted approximately 45 minutes and took place in a private room in the sports club, the participant’s home, or a similar private venue between April and May 2019.

**Semistructured interview guide used in this study.**

Topic 1: Rehabilitation experiences

- 1.1 Please talk me through your process of rehabilitation, from the time of the surgery until now.
- 1.2 Can you describe your awareness of how the operation would impact you, both short and long term?
- 1.3 Tell me about a time throughout the rehabilitation process that you found difficult.
- 1.4 What were your expectations of the physiotherapy care you would receive? How did they match the reality?
- 1.5 How well-equipped did you feel to do the postoperative rehabilitation at home? How confident did you feel doing the exercises?
- 1.6 Were you given exercise instructions and information? How?
- 1.7 Is there anything that might have helped you during rehabilitation after the surgery?

Topic 2: Digital technologies in breast cancer rehabilitation

- 2.1 Did you use any modern technologies to help you during your rehabilitation?
- 2.2 Do you use any technologies for your general health and well-being?
- 2.3 When discussing your experiences of physiotherapy, you mentioned [insert problem]. If there was a piece of technology to help you with this issue, what would it do?

### Data Analysis

The interviews were transcribed verbatim and anonymized transcripts were imported into the qualitative data analysis management software NVivo (QSR International). The lead researcher applied a thematic analysis with a semantic, mixed inductive and deductive approach to analyze the data, following the process outlined by Braun and Clarke [56]. After a data familiarization stage, the data was categorized into codes, which contained conceptually similar ideas or actions. Applying an iterative process, the relationships between codes were analyzed and themes were formed by grouping related codes together. A second researcher who specializes in qualitative data analysis (TK) applied the same inductive thematic analysis process to review and code extracts from 7 transcripts. The two researchers compared codes and themes, and resolved any differences in coding through discussion. Data saturation was determined when no new themes and relationships among the interview data were found [62]. The lead researcher then performed a final revision of the thematic analysis to ensure that the themes accurately reflected the content of the data set and to screen for internal and external heterogeneity of themes [63]. Final themes were agreed, defined, and named by the two researchers. Illustrative quotes for each code and theme were identified. Participants did not receive a copy of the transcript or provide feedback on the findings.

## Results

### Participant and Surgery Characteristics

In total, 10 women participated in the semistructured interviews. Participant characteristics are summarized in Table 1. Information on the participants’ surgeries and physiotherapy received are described in Table 2. Postoperative physiotherapy services mainly consisted of a single postoperative assessment, which for the purposes of this study will be defined as a single visit by the physiotherapist to review the patient, teach postoperative exercises, and assist in mobility or other needs. Those who had physiotherapy arranged as a follow-up (n=2) or a later referral (n=2) reported the experience to be very helpful and educational, with supportive and highly skilled physiotherapists.

**Table 1:** Participant characteristics.

Participant characteristic		Participants, n
Female		10
**Age (years)**		
	35-44	1
	45-54	6
	55-64	2
	65-74	1
**Ethnicity**		
	White (Irish)	10
**Highest level of education**		
	Trade/technical/vocational training	1
	Diploma	1
	Some university	1
	Bachelor’s degree	3
	Master’s degree	3
	Doctorate	1
**Employment status**		
	Unable to work	2
	Employed	5
	Self-employed	1
	Temporary retirement	1
	Retired	1
**Approximate annual household income (€)**		
	30,000-50,000	3
	50,000-75,000	4
	75,000-100,000	2
	>100,000	1
**Marital status**		
	Single	1
	Married or domestic partnership	7
	Separated or divorced	2

**Table 2:** Characteristics of breast cancer surgery for each participant^a^.

Type of breast surgery		Physiotherapy received after surgery	Extent of axillary surgery	Time since surgery (years)	Public or private health care
**Participant 1**					
	Bilateral mastectomy	Postoperative assessment	Axillary clearance	4	Public
	Axillary clearance				
**Participant 2**					
	Lumpectomy	Postoperative assessment	Axillary clearance	2.4	Private
	Lumpectomy and axillary clearance				
**Participant 3**					
	Axillary clearance	Unknown^b^	Axillary clearance	1.2	Public patient in private hospital
	Mastectomy	Unknown			
	Reconstruction (type unknown)	Seen daily in hospital			
**Participant 4**					
	SLNB^c^ and lumpectomy	No	Axillary clearance	4	Private
	Axillary clearance	Participant unsure			
	Mastectomy	Postoperative assessment			
	DIEP^d^ flap reconstruction	Postoperative assessment and out-patient follow-up; later referred by oncologist			
**Participant 5**					
	Bilateral mastectomy	Postoperative assessment and later self-referral	Axillary clearance	3.4	Public patient in private hospital
**Participant 6**					
	SLNB	Postoperative assessment	SLNB	2.5	Private
	Lumpectomy				
**Participant 7**					
	Mastectomy with reconstruction	Postoperative assessment	Unknown	4	Public
**Participant 8**					
	Mastectomy with DIEP flap reconstruction	Postoperative assessment	Unknown	2.2	Private
**Participant 9**					
	Mastectomy with implant reconstruction	Postoperative assessment	Unknown	1.5	Private
**Participant 10**					
	SLNB	Postoperative assessment and out-patient follow-up	Axillary clearance	1	Private
	Mastectomy and axillary clearance				

^a^To preserve anonymity, participants are not listed in order and participant numbers do not correspond to those used throughout the manuscript.

^b^“Unknown” refers to missing data that was not gathered during the interviews [64].

^c^SNLB: sentinel lymph node biopsy.

^d^DIEP: deep inferior epigastric perforators.

### Topic One: Rehabilitation Experiences

Under the topic of “rehabilitation experiences,” four main themes were identified: acute and long-term consequences of surgery; unmet needs and lack of support; self-driven rehabilitation; and visions for high quality rehabilitation.

#### Theme 1: Acute and Long-Term Consequences of Surgery

Participants experienced short-term and long-term consequences of the surgery, which impacted their function and quality of life. Long-term upper limb dysfunction was described by Participant (P) 6: “This arm is definitely still a bit weaker. If I go to pick things up, picking up groceries or whatever, I don't have the same strength in this arm.” As well as physical impairments of stiffness and weakness, participants experienced feelings of overwhelm, fear, and anxiety. The prospect of developing side effects of surgery could be worrying; P8 stated, “I was terrified of lymphedema.” Most participants were surprised by how they felt emotionally after the surgery, and report not being prepared for what to expect:

I had no idea that there would be such life changing things going on in my body. You kind of think, you’ll have your surgery…and then life will go back to normal, but it doesn't.

P6

Secondary musculoskeletal complications developed for several participants, such as frozen shoulder, bursitis, and postural problems. P2 reported how she could not move her upper limb into the required position to receive radiotherapy:

The first time around, I developed a frozen shoulder. This was very evident when I was going for my radiotherapy, I couldn’t do it.

#### Theme 2: Unmet Needs and Lack of Support

Participants were not always aware of the importance of physiotherapy and rehabilitation and several felt that there was a lack of emphasis on rehabilitation throughout their hospital stay:

I mean I was told, but it wasn't heavily enforced and it wasn't really stressed. I don't feel that it was high priority.

P5

As demonstrated in Table 2, most participants only had one assessment with the physiotherapist, with no follow-up appointment. P2 explained:

Your physiotherapist comes to you in the ward, goes through the exercises with you, but other than that, there isn’t any real physio support, you do the exercises yourself.

This was felt to be insufficient, especially if concerns arose after this assessment, as P6 expressed: “I didn't really feel like I had a physiotherapist that I could ring up and talk to.” There were also challenges with how the HEP was provided. The sheet of paper containing descriptions of the exercises was carefully minded by some participants, but others, including P4, didn’t take the same approach: “you don't know where you have left it half the time.” Several participants felt that the physiotherapy assessment took place too soon after the surgery. P10 explained: “I was probably still on morphine, so I probably wasn’t paying enough attention.”

As a result of the above unmet needs, P1 reported feeling uncertain about how to perform the exercises:

I didn't even know whether I was doing it right or not, you know, sitting on the bed supposed to be doing this or this, or whatever, and I didn't bother then.

P1

Additionally, there were difficulties with the postoperative self-management advice that they had received from the hospital. The information was “very unstructured and informal” [P5] and two participants felt it was too focused on what not to do: “don’t do this, don’t do this; it wasn’t reassuring to me in any sense” [P4]. Upon discharge from hospital, treatment side effects restricted participants’ abilities to do the HEP; they reported feeling “quite weakened” from chemotherapy [P8], that radiotherapy “affected my skin and it made the stretching very sore” [P10] and that fatigue was “the biggest impediment to doing (the HEP)” [P4]. Financial barriers prevented two participants from arranging private physiotherapy appointments. Time and scheduling were additional barriers to home rehabilitation, as was a fear of movement:

I think a woman is naturally terrified of it and afraid that those stretch things are going to, you know, hurt, so, I think there is just a barrier.

P8

At a time when professional advice and support was much needed, participants felt alone and unsupported:

Certainly, post-surgery you feel quite isolated, whereas, in chemo you are meeting oncology nurses all the time, but post-surgery…you are on your own.

P8

Having little contact with health care professionals was especially difficult when participants experienced unexpected side effects, as P8 reported: “I found it quite shocking and terrifying…essentially I had nobody to turn to.”

#### Theme 3: Self-Driven Rehabilitation

A desire and motivation to recover was expressed by all participants. Despite the unmet rehabilitation needs and barriers to home rehabilitation, 6 participants expressed they had had a strong internal motivation which led them to persevere with the HEP. P4 asserted: “if you say to me that this is what you need to do to get better, I'll do it.” Another motivating factor was personal experience of upper limb dysfunction:

There is no question about it. I felt if I didn't stretch my arm…it would be very stiff and, and very, very sore.

P6

When participants did have follow-up physiotherapy appointments, this sense of accountability was an external motivating factor. Participants were proactive in fulfilling their own rehabilitation needs; for example, they sourced information, support and advice as required:

It's hard to do nothing, therefore whatever is at hand, you will look up, research and investigate or speak to…people like to feel like they are being pro-active about their own recovery.

P5

There was a strong role for peer support, and speaking to other individuals with breast cancer was an effective way to gain knowledge, advice and assistance:

It was very rarely involved interacting with professionals, it was almost exclusively peer to peer, asking other women, what to do about lymphoedema, what to do about, when to wear a sleeve?

P5

The holistic benefits of exercise were mentioned by all participants. Some participants tried new forms of exercise to address surgery-related upper body dysfunction, including yoga, Pilates, and going to the gym:

My posture was horrendous, I was just completely rounded, you know, to hide… So now, through the Pilates…my posture is greatly improved.

P2

Overall, 3 participants reported that they did not engage in exercise prior to surgery, but continued because they saw the benefits of the particular activity. P1 stated that, “There is a whole new me, from being a couch potato all my life, to doing this.” Additionally, 8 participants discussed the social and psychological benefits of exercise, which ranged from having improved confidence and feeling calmer, to having the opportunity to get fresh air and make new friends. Regarding the sports club, participants spoke of finding “a great camaraderie” [P3] and of “meeting people who have been through something similar, you can all relate” [P4]. P10 expressed gratitude for the club and her experience as a member of that community:

I also think that was the silver lining to the whole experience ‘cos now I have a whole life around that, and a whole lot of new friends, so that has been brilliant.

#### Theme 4: Visions for High Quality Rehabilitation

Discussions of good experiences with health care professionals and recommendations for service improvements provided insights into patient preferences for breast cancer rehabilitation. Participants appreciated proactive care and patient education. P6 described a valuable interaction with her physiotherapist: “she wasn't forceful but she made it clear that if you don't do your exercises you will be left with less movement.” All participants recommended more access to physiotherapy services, especially follow-up assessments after discharge from hospital:

I would have liked to, maybe, have gone through the exercises that I was trying to do, check that I was doing them properly.

P3

Several participants recommended providing patients with more information about what to expect after the surgery, regarding diet, exercise, and breast prostheses. One participant suggested this could be done through a physiotherapist-led preoperative information session, while another requested a postoperative support group:

A support group for people, for the after-effects of surgery and everything else, which would include the physiotherapist plus maybe other things as well.

P6

### Topic Two: Technology

The experiences of the participants with mHealth technology and the features that participants would like to see in an mHealth tool for breast cancer rehabilitation were the two distinct themes from this portion of the interviews.

#### Theme 1: Experience with Digital Technology for Health

The digital technologies used by participants for health and rehabilitation are detailed in Table 3. None of the participants used a breast cancer–specific mHealth tool; instead, participants applied technology for the general population to meet their cancer rehabilitation needs. Only 1 participant did not use any digital technology for health, stating that, “I think here we are seriously looking at an old age thing” [P3].

Technology played two main roles: first, it acted alongside health care professionals, for example to help patients understand information provided by the hospital: “[The terminology] was all new language to me, so when I came home I did google all that” [P1]. Second, it compensated for unmet needs in health care provision, for example to provide exercise advice: “I would do Pilates, I would watch an app to do that” [P6]. Overall, 6 participants used activity monitors, and they perceived them as good motivators to be physically active, providing a sense of achievement when participants reached their goals. Some disadvantages of mHealth were expressed, such as feeling overwhelmed by the volume and content of information: “you can really live in cancer-land” [P6]. Concerns about data privacy were raised: “What happens if [your personal information] somehow gets into the wrong hands?” [P7]. Additionally, it was acknowledged that technology cannot replace the role of the human in health care:

As much as you want apps or websites, you also need the human bit, you need them all.

P10

**Table 3:** Participants’ use of digital technology and mHealth.

Technology characteristic		Participants, n
**Smartphone owner**		
	Yes	10
**Uses smartphone apps or wearables in daily life**		
	Yes	10
**Used mHealth tool designed for postoperative rehabilitation**		
	No	10
**Used or uses digital technology for general cancer rehabilitation issues^a^**		
	Activity monitor: wearable	5
	Activity monitor: smartphone	4
	Guided exercise app	2
	Mindfulness app	2
	Cancer-themed podcast	1
	Online breast cancer magazine	1
	Nothing	1
**Used or uses digital technology for health information seeking**		
	Yes	5
	Yes, occasionally	2
	Not sure	2
	No	1

^a^Some participants used more than one digital technology for rehabilitation issues.

#### Theme 2: Requirements for an mHealth System

All participants stated that they would have liked to have tried out an mHealth system during home rehabilitation if such a system had been available. Throughout the discussion, participants suggested features they would like to see in such a system (Table 4). These features largely focused on exercise information and guidance. In total, 4 participants suggested having a visual aid in the form of a physiotherapist or a biofeedback avatar performing the exercises alongside them. They felt that an avatar should resemble someone who could have had breast cancer treatment, but not someone currently in treatment:

I don't think you would need anybody bald, you wouldn't want, necessarily, that reminder. Maybe not somebody with gorgeous locks, short hair would be fine.

P5

A desire for both audio and visual exercise biofeedback was expressed by P9: “If there is going to be an audio, having something visual as well, it’s there if I wanted to check, ‘Yeah, I am doing this correctly.’” It was important to many that the system should be provided from the hospital: “if it's through the hospital, then, you know, it's got that legitimacy” [P5]. A record of exercise performance was a popular feature, not only because it would provide feedback to patients, but because it would remotely provide feedback to the physiotherapist as well:

I would like to know that…somebody had taken an interest and a follow-up in what I am doing…and then they would be able to intervene if they saw I wasn’t doing it properly.

P1

Regarding the style of the system, there was a strong preference for a “less is more” approach, with concise information that is specific to the patient’s own situation:

Your concentration is shot after cancer when you are having treatment…You need to ask a question and get an answer.

P2

There was a preference toward content that is positive and life-affirming, delivered with an encouraging and reassuring tone:

I think maybe some encouragement because it's such a difficult time, not just 'do this,' but 'how are you finding it?' You know, 'how's your day?'

P5

**Table 4:** Desired features in an mHealth home rehabilitation system: participant suggestions and frequency of suggestion.

Feature	Suggestion frequency, n
Information on exercises	8
Record of exercise sessions	6
Reminders	5
Motivational messaging	4
Exercise videos	4
Personalization of content	3
Contact with physiotherapist	3
Interact with other patients	2
Postoperative information	2
Goal setting	2
Repetition counter	2
Exercise progression generator	1

## Discussion

### Principal Findings

This study presented detailed insights into the physical rehabilitation experiences and unmet needs of 10 women after surgery for breast cancer. It has highlighted the barriers to home rehabilitation and presents participants’ visions for high-quality rehabilitation. Through this, the role of an mHealth system with biofeedback features to reduce barriers and improve quality of care can be seen. Additionally, this study explored patient preferences and requirements for such a system. Under the topic of “rehabilitation,” themes covering the experiences of acute and long-term consequences of surgery; participants’ unmet needs and feelings of lack of support; the motivation and need to take the lead in one’s own rehabilitation; and participants’ visions for high-quality rehabilitation were identified. Under the “technology” topic, the sample’s experience with digital technology and mHealth were outlined, and key requirements for an mHealth home rehabilitation support system were identified.

### Comparison with Prior Work

The findings regarding the acute and long-term consequences of breast cancer surgery were wholly in line with previous research. Throughout the “rehabilitation” section of the interview, participants reported a desire for increased access to physiotherapy services, both before and after surgery, and at later stages of treatment. Alongside clinical practice guidelines, other research with patient populations also supports these recommendations for routine preoperative and follow-up physiotherapy services [18,65,66]. Many participants had extensive breast surgery and axillary clearance (Table 2), which are risk factors for the development of lymphedema [67] and arm and shoulder impairments [68], indicating that this sample had substantial rehabilitation needs from the time of surgery onward. All participants experienced postoperative upper limb dysfunction, which for some resolved after the acute postoperative period, but, as reported by Ewertz et al [7], these symptoms could last long after treatment completion. As also described by Easley et al [69] in their analysis of interviews with young breast cancer survivors, at the time of treatment many participants were not made aware of rehabilitation services or the importance of rehabilitation. Easley et al stated that their participants also experienced a lack of support and had “no place to turn for help.” In work by Harder et al [70] and Lee et al [71], participants often felt that the perioperative information they received was insufficient and poorly timed; participants in this study shared this perspective. The recurrence of these themes throughout literature that spans a decade suggests that improvements in accessing rehabilitation services are not happening quickly enough, and that innovative methods of improving access to care are needed.

### Implications of Findings

The unmet needs described by participants in this study include a need to be educated in advance about the impact of the surgery and the importance of rehabilitation; a need for more physiotherapy services; a need for timely and clearly presented information regarding postoperative care; a need for more support on discharge and the ability to contact the appropriate health care professional when concerns arise. These unmet needs highlight areas in which improvements to care could be made, either through traditional service development methods or through the use of technology. An mHealth system, such as an app, could assist in these improvements by being a portable source of postoperative support by providing information, prescribed exercises, and a method of remote contact with a physiotherapist. Improved clinical outcomes have been demonstrated by combining a standard postoperative HEP with an mHealth app and biofeedback technology [35,72]. Additionally, Lambert et al [73] report that people with musculoskeletal conditions self-report adhering to their HEP better with an app than with a paper handout.

An important finding of this study was the participants’ high levels of motivation to recover after surgery. Although adherence to HEPs has been found to be low in many populations [13,74,75], it is not necessarily the case in this population. Essery et al [76] report that self-motivation is a positive predictor of adherence to home rehabilitation. Although many participants reported doing their exercises “religiously,” there was uncertainty regarding the exercises and barriers to performing the HEP. This may have led participants to perform poor-quality rehabilitation or to stop doing the HEP prematurely. Improving motivation to exercise should therefore not be a primary goal of mHealth in this context; instead, the focus should be on improving the quality of rehabilitation.

No participants used mHealth technology for their postoperative rehabilitation. This reflects the lack of digital technology specifically designed for this period of breast cancer care and a lack of awareness of available apps, rather than a lack of desire to use mHealth, as all participants stated they would have used a suitable mHealth tool if it was available. Older age was a limiting factor for 1 participant for use of digital technology in daily life, but it was not a barrier to her desire to use mHealth. This is consistent with research by Abelson et al [77] which found that older adults are as willing as younger adults to engage with mHealth. Technologies with the capacity for objective, detailed in-clinic assessment are emerging, such as the Kinect (Microsoft Corp) reachable workspace system, as applied in a breast cancer population by Uhm et al [78], and a smartphone-based fatigue test developed by Cuesta-Vargas et al [79]. As a key stakeholder in these technologies, the openness of patients to the use of effective, helpful technologies is essential. After the acute stage of recovery, participants often used non–cancer-specific consumer technologies for physical activity and mental well-being. However, these may not meet the needs of individuals with breast cancer, as they do not consider fatigue, upper limb dysfunction, and other disease-specific considerations [80]. Zhou et al [81] developed a program using WeChat to provide physical, psychological, and social guidance after breast cancer surgery. Although this did not support the user through biofeedback features, it provided an opportunity to communicate remotely with a nurse or doctor from the breast surgery team at a specific time of day.

Overall, participants saw value in an mHealth tool to support patients during home rehabilitation after breast cancer surgery. They requested a system which focuses on providing exercise information, feedback on progress, and guidance in an audiovisual manner. This is echoed in a 2019 exploration of breast cancer survivors’ preferences for mHealth physical activity interventions by Phillips et al [80], who found that exercise progress feedback and exercise scheduling were highly desirable features. Several of the suggested features for an mHealth system can be categorized as biofeedback features (record of exercise sessions, repetition counter, motivational messaging), and these can be enabled through the use of a wearable external sensor. This sensor data could also be applied to optimize additional feedback-related features (contact with physiotherapist, interact with other patients, goal setting, exercise progression generator). External sensors are increasingly being used in cancer care for both research and clinical purposes [30,82]. They can be facilitators to exercise, as found by Kokts-Porietis et al [55] who used wrist-worn activity monitors in a physical activity intervention with breast cancer survivors.

Finally, it was important to participants that an mHealth system would be provided by the hospital or a health care provider, and that it would include a remote connection with the physiotherapist. Chandra et al [83] found that physiotherapy patients mainly felt “motivation by support when their physiotherapist could view their exercise record, but were also unsure if physiotherapists would actually view it. Physiotherapists were cautiously positive about remote communications, but had reservations regarding increased workload. This feature is clearly valuable to the patient, but should be developed closely with the physiotherapist to ensure it is appropriate for all stakeholders. Additionally, participants were keen to retain some face-to-face contact with the physiotherapist, and therefore an mHealth system in this context should be provided by the physiotherapist as an adjunct to regular care.

### Study Limitations

Breast cancer is a disease which affects 1 in 8 women across their lifetime, and therefore the experiences of women with breast cancer are incredibly varied and impossible to capture in a single study. The themes presented strongly across the participants and therefore appear to be highly representative of the population [62]. Specific limitations of our sample are that they were self-selecting and were actively engaged in a sports team after their treatment. Although participants were not all habitual exercisers prior to their diagnosis, they may have inherent positive attitudes toward exercise performance. Due to limited human resources, a full analysis of the transcripts was performed by only one author. A second reviewer analyzed sections of the transcripts to validate the lead researcher’s analysis, and all authors reviewed and discussed the themes, codes, and quotes.

### Conclusions and Future Work

An mHealth intervention has the potential to support patients during rehabilitation after breast cancer surgery. Understanding unmet needs of users and their perspectives on mHealth at an early phase of development enables the co-design of a user-friendly and relevant system. A thematic analysis of interviews with breast cancer survivors reflecting on their rehabilitation experiences demonstrates that, while participants were motivated to exercise, a lack of support on discharge and a lack of emphasis on rehabilitation led to uncertainty during rehabilitation and feelings of being alone and unsupported. Participants stated they would have welcomed an mHealth support tool that was reliable, provided by the hospital, and focused on rehabilitation support. The results of this analysis support a drive for improvements in breast cancer rehabilitation, which can be led by technological and nontechnological means. The impact of rehabilitation promotion and facilitation of access to postoperative physiotherapy services on rehabilitation outcomes should be investigated. These results provide motivation and rationale to develop an mHealth system with biofeedback that strives to improve outcomes for these often-undertreated consequences of breast cancer treatment.

## Abbreviations

eHealth: electronic health
HEP: home exercise program
mHealth: mobile health
P: participant
ROM: range of motion
